# Design and Characterization of a Recombinant *Brucella abortus* RB51 Vaccine That Elicits Enhanced T Cell-Mediated Immune Response

**DOI:** 10.3390/vaccines10030388

**Published:** 2022-03-03

**Authors:** Mahdieh Sarmadi, Azam Gheibi, Hossein Khanahmad, Mohammad Reza Khorramizadeh, Seyed Hossein Hejazi, Noushin Zahedi, Hamidreza Mianesaz, Khosrow Kashfi

**Affiliations:** 1 Department of Genetics and Molecular Biology, School of Medicine, Isfahan University of Medical Sciences, Isfahan 81746-73461, Iran; m.sarmadi@viravaccine.com (M.S.); zahedi.nsh@gmail.com (N.Z.); h.mianesaz@gmail.com (H.M.); 2 Department of Biotechnology, School of Advanced Technologies in Medicine, Tehran University of Medical Sciences (TUMS), Tehran 14177-55469, Iran; khoramza@tums.ac.ir; 3 Biosensor Research Center, Endocrinology and Metabolism Molecular-Cellular Sciences Institute, Tehran University of Medical Sciences (TUMS), Tehran 14117-13139, Iran; 4 Department of Parasitology and Mycology, School of Medicine, Isfahan University of Medical Sciences, Isfahan 81746-73461, Iran; hejazih12@gmail.com; 5 Department of Molecular, Cellular, and Biomedical Sciences, Sophie Davis School of Biomedical Education, City University of New York School of Medicine, New York, NY 10031, USA; 6 Graduate Program in Biology, City University of New York Graduate Center, New York, NY 10016, USA

**Keywords:** *Brucella abortus*, RB51 strain, listeriolysin O (LLO), BAX and SMAC apoptotic proteins, Th1 immune response

## Abstract

*Brucella abortus* vaccines help control bovine brucellosis. The RB51 strain is a live attenuated vaccine with low side effects compared with other live attenuated brucellosis vaccines, but it provides insufficient protective efficacy. Cell-mediated immune responses are critical in resistance against intracellular bacterial infections. Therefore, we hypothesized that the listeriolysin O (LLO) expression of *Listeria monocytogenes*, BAX, and SMAC apoptotic proteins in strain RB51 could enhance vaccine efficacy and safety. *B. abortus* RB51 was transformed separately with two broad-host-range plasmids (pbbr1ori-LLO and pBlu–mLLO-BAX-SMAC) constructed from our recent work. pbbr1ori-LLO contains LLO, and pBlu–mLLO-BAX-SMAC contains the mutant LLO and BAX-SMAC fusion gene. The murine macrophage-like cell line J774A.1 was infected with the RB51 recombinant strain containing pBlu-mLLO-BAX-SMAC, RB51 recombinant strain containing LLO, and RB51 strain. The bacterial cytotoxicity and survival and apoptosis of host cells contaminated with our two strain types—RB51 recombinants or the parental RB51—were assessed. Strain RB51 expressing mLLO and BAX-SMAC was tested in BALB/c mice and a cell line for enhanced modulation of IFN-γ production. LDH analysis showed that the RB51-mLLO-BAX-SMAC and RB51-LLO strains expressed higher cytotoxicity in J774A.1 cells than RB51. In addition, RB51 recombinants had lower macrophage survival rates and caused higher levels of apoptosis and necrosis. Mice vaccinated with the RB51 recombinant containing mLLO-BAX-SMAC showed an enhanced Th1 immune response. This enhanced immune response is primarily due to bacterial endosome escape and bacterial antigens, leading to improved apoptosis and cross-priming. This potentially enhanced TCD_8_^+^- and T cell-mediated immunity leads to the increased safety and potency of the RB51 recombinant (RB51 mLLO-BAX-SMAC) as a vaccine candidate against *B. abortus*.

## 1. Introduction

The genus *Brucella* comprises Gram-negative intracellular bacterial pathogens that cause brucellosis, resulting in abortion in domestic animals and causing considerable economic losses in the world livestock sector and undulant fever in humans [1,2]. Cattle are a typical host for *Brucella abortus*, although *Brucella abortus* can cause infections in sheep, goats, pigs, bison, buffalo, horses, elk, and many other animal species. Animal vaccination, especially in endemic areas, is the most economical and critical way to control brucellosis. Animal vaccination would also minimize potential human infections [3,4]. A small number of live attenuated vaccines are available for animal immunization against brucellosis [5]. However, these have significant limitations, including interference with diagnostic tests through anti-LPS antibody induction, human pathogenicity, persistent infection in vaccinated animals, virulence recurrence risk, secretion into milk, and abortions in pregnant animals even at a single dose and without long-lasting protection [6,7]. In many countries, the *B. abortus* RB51 rough strain live attenuated vaccine is used instead of *B. abortus* S19 [8]. This vaccine is a spontaneous mutant obtained by sub-culturing the virulent strain *B. abortus* 2308 on a medium containing rifampicin and penicillin [9]. There are limitations to the immunization efficacy of RB51 depending on the challenge, with no absolute protection achieved with this vaccine [10]. Thus, there is an urgent need to develop effective vaccines that could prevent brucellosis infection altogether.

*Brucella* uses particular mechanisms to prevent the *Brucella*-containing vacuole (BCV)–lysosome fusion and, as a result, survives for long periods and replicates in the endoplasmic reticulum (ER) without detection by the innate immune system and minimization of the adaptive immune response [11,12]. As a result, pathogen antigens in the phagosome are less recognized by the immune system, especially cell-mediated immunity. Cell-mediated immunity plays a critical role in resistance to intracellular bacterial infections [13]. *Listeria monocytogenes* enables the escape of the host phagosome, utilizing listeriolysin (LLO), a pore-forming cytolysin, representing a unique mechanism to facilitate antigen presentation of *Listeria* antigens to the immune system. It seems that the transfer of this escape function strategy to other intracellular bacteria such as *Brucella* leads to a more pronounced major histocompatibility complex (MHC) class I presentation [14]. Thus, the cell-mediated immune system has better access to the antigens of pathogens [14]. Consequently, a more effective response is generated by the immune system.

Bax is a core regulator of the mitochondrial apoptotic pathway. This pathway promotes mitochondrial outer membrane permeabilization and allows the release of proapoptotic factors such as SMAC/DIABLO from the mitochondria into the cytosol to activate the caspase cascade, leading to cell death [15,16]. We hypothesized that the expression of the biologically active LLO and BAX-SMAC apoptotic proteins in the RB51 strain could improve the access of *Brucella* antigens to the immune system. Endosomal escape of the RB51 strain and its antigens using LLO can lead to cross-priming and apoptosis in the infected macrophage. Displaying antigens by the dendritic cells after apoptosis of the macrophages could enhance the induction of TCD_8_^+^- and Th1-type immune responses and lead to higher vaccine efficacy and safety.

## 2. Materials and Methods

This study was approved by the Ethics Committees of Isfahan University of Medical Science, Isfahan, Iran (ethic code IR.MUI.REC.1396.3.443). Experiments were performed under biosafety conditions in animal laboratories, following the committee’s guidelines. 

### 2.1. Bacterial Strains

The *Escherichia coli* TOP10F’ strain was grown at 37 °C in Luria–Bertani (LB) broth or agar (Merck, Frankfurt, Germany) containing 80 μg/mL of tetracycline.

The *Brucella abortus* RB51 strain was grown at 37 °C in trypticase soy agar (TSA) or trypticase soy broth (TSB) (Quelab, QC, Canada) supplemented with 50 μg/mL of rifampin [17].

### 2.2. Cell Line

The J774A.1 (ATCC product TIB-67) mouse BALB/c monocyte–macrophage cell line, derived from a tumor in a female BALB/c mouse, was grown under conventional cell culture conditions in a complete medium (c-DMEM) consisting of Dulbecco’s modified Eagle’s medium (DMEM; ATCC) supplemented with 10% heat-inactivated fetal bovine serum at 37 °C in a 5% CO_2_ atmosphere [18].

### 2.3. Construction of Recombinant Brucella abortus RB51 Strains Expressing LLO and mLLO-BAX-SMAC

The origin of replication of the pBBR1 plasmid that can be replicated in *Brucella* spp was separated and ligated to the pre-prepared pBGGT_LLO plasmid, and pBBR1ori_LLO (contains the LLO gene with the groE promoter and the bcsp31 signal peptide from *Brucella* spp.) was constructed [19,20,21]. To have optimal pH activity inside the host cells, we used an LLO gene in which leucine 461 was changed to threonine, as this is responsible for LLO’s optimum acidic pH [22]. The gene construct containing the mutant LLO-BAX-SMAC (mLLO-BAX-SMAC) was obtained from GeneCust (Luxembourg) and used for synthesizing and subcloning in *Nco*I and *BglI*I sites of pBlue-BHori. The resulting plasmid (pB-mLLO-BAX-SMAC) contains the mutant LLO gene (L461T) with the groE promoter and the murine BAX-SMAC gene with the chloramphenicol promoter. Initially, *Escherichia coli* TOP10F’ was transformed with pB-mLLO-BAX-SMAC and pBBR1ori_LLO plasmids separately and grown on an LB agar plate containing ampicillin or kanamycin at a concentration of 100 and 25 mg/mL. Positive clones of *E. coli* containing pB-mLLO-BAX-SMAC or pBBR1ori_LLO plasmids were selected and cultured in LB broth, and plasmid extraction was performed using a Solgent plasmid extraction kit (Solgent, Daejeon, South Korea). Electroporation was used to introduce pB-mLLO-BAX-SMAC and pBBR1ori_LLO plasmids into *B. abortus* strain RB51 separately. These bacteria were grown in TSB at 37 °C for 48 h. The cells were chilled on ice for 30 min and centrifuged at 5500 rpm for 7 min. The cell pellet was resuspended in an equal volume of sterile, cold deionized water and centrifuged as described above. The bacteria were washed twice with water, and in the last washing step, 10% glycerol was used instead of water; the cells were resuspended in a 1/500 volume of 10% glycerol and kept on ice. Dialyzed DNA plasmids (pB-mLLO-BAX-SMAC or pBBR1ori_LLO) were added and pipetted in sterile electroporation cuvettes with electrode gaps of 0.2 cm (BioRad Laboratories, Richmond, CA, USA). A Gene Pulser transfection apparatus (BioRad Laboratories) at a 25 uF and 2.5 kV setting with the pulse controller set at 400 Ω was used for the transformation. Immediately after electroporation, 1 mL of TSB was added to the bacteria, and the bacteria were cultured at 37 °C for 24 h. The bacteria were grown on TSA plates supplemented with 50 ug of ampicillin or 25 ug of kanamycin per mL. After 4 days, several colonies of strain RB51 containing the different plasmids were obtained from a TSA plate [23]. The presence and stability of the cloned plasmids in vitro were verified after 50 successive passages.

### 2.4. Confirmation of Transformation and Western Blotting

BAX-SMAC and mutant LLO expression was assessed in the RB51 recombinant strain using SDS-Western blot analyses and evaluation of LLO hemolytic activity [24,25].

Sodium dodecyl sulfate-polyacrylamide gel electrophoresis (SDS-PAGE) was performed to detect BAX-SMAC and mutant LLO expression according to standard procedures [26]. Briefly, cultures of *B. abortus* RB51, RB51LLO, and RB51-mLLO-BAX-SMAC grown on TSB were harvested, and samples were prepared by diluting the bacterial suspensions in Laemmli sample buffer and heating. Samples were re-suspended in SDS loading dye and electrophoresed on a 12% SDS-PAGE gel. After electrophoresis, proteins separated by SDS-PAGE were electro-transferred onto a nitrocellulose membrane. The nitrocellulose membrane was incubated and blocked with 2% bovine serum albumin solution. After three washes, a horseradish peroxidase-conjugated anti-histidine tag was added. After incubation for 2 h at room temperature, the membrane was washed three times and covered with the peroxidase substrate. 

### 2.5. LLO Hemolytic Activity

The hemolytic activity of LLO and mutant LLO was evaluated by lysis of sheep erythrocytes on a plate. Briefly, RB51 and both our RB51 recombinants (RB51-LLO and RB51-mLLO-BAX-SMAC) were cultured on TSA plates containing 5% sheep red blood cells supplemented with 50 μg/mL of rifampin or rifampin ampicillin per mL and 25 μg/mL of rifampin kanamycin at 37 °C for 48–72 h. LLO and mutant LLO expression by the two different RB51 recombinants was evaluated by hemolytic activity on a plate compared with the parental RB51 [25].

### 2.6. Lactate Dehydrogenase (LDH) Assay

LDH release was used to evaluate the cytotoxicity of the two different RB51 recombinants. About 5 × 10^4^ J774A.1 cells were seeded in each well of a 96-well plate and tested in triplicates. Each group of cells was infected with 3 × 10^9^ RB51 and RB51 recombinants. In the negative control group, only the J774A.1 macrophage cell line was added. At 6 and 24 h post-infection, the culture supernatants and cell lysates were collected and assessed for LDH activity using a CytoTox 96 non-radioactive cytotoxicity assay kit (Promega, Madison, WI, USA) according to the manufacturer’s instructions [27].

### 2.7. Evaluation of Brucella Survival

About 2.5 × 10^5^ J774A.1 macrophage cells were seeded in 24-well plates and incubated at 37 °C. After 24 h, these cells were infected with about 3 × 10^9^ of RB51 and RB51 recombinant strains, separately [28,29]. The intact cells were used as a control. The plates were centrifuged at room temperature for 5 min at 300× *g* for penetration and effective phagocytosis. All plates were incubated at 37 °C and 5% CO_2_. After 1 h, the cells were washed three times with phosphate-buffered saline (PBS) and incubated in fresh DMEM supplemented with 50 μg/mL of gentamicin to kill extracellular bacteria. To assess the intracellular survival of *Brucella*, 24 h after infection, the cells were lysed with 1 mL of sterile 0.1% (*v*/*v*) TritonX-100. The number of colony-forming units (CFU) was measured by plating a series of dilutions on TSA plates containing specific antibiotics and sheep red blood cells to determine our plasmids’ existence by different antibiotic resistances and secretions of the LLO protein. Additionally, the plasmids extracted from the recovered bacteria were tested for digestion mapping [30].

### 2.8. Evaluation of Programmed Cell Death

J774A.1 macrophage cells were seeded in six-well plates (1 × 10^6^ cells/well) and infected with 3 × 10^9^ RB51 and two different RB51 recombinants, separately. Infected cells were stained with Annexin V and propidium iodide using an FITC Annexin V kit (San Diego, CA, USA) 24 and 48 h after infection and incubated at room temperature for 20 min. Flow cytometry was used to detect apoptotic and necrotic macrophage cells [31]. 

### 2.9. Animal Testing for Clearance Experiments

Three groups of six-week-old female BALB/c mice (N = 14 per group) (supplied by the Pasteur Institute of Iran) were injected intraperitoneally with ~4/5 × 10^8^ bacteria (strain RB51 and RB51 recombinant containing mLLO-BAX-SMAC) [28,29]. Saline alone was used in the negative control group. Three weeks post-inoculation (p.i.), 4 mice from each group were sacrificed, their spleens were aseptically removed, homogenized in phosphate-buffered saline (PBS), and then cultured on TSA medium, and CFU numbers were determined to assess clearance [32]. Recovered bacteria from spleens were spread on TSA containing specific antibiotics and sheep red blood cells for determining our plasmids’ existence by different antibiotic resistances and secretions of the LLO protein. Additionally, plasmids extracted from recovered bacteria were tested for digestion mapping.

### 2.10. Cytokine Quantitation in Immunized Mice

Blood samples of five mice from each group were collected 4 and 6 weeks p.i.; IFN-γ was measured using an ELISA kit (Mouse IFN-γ Biolegend-USA). Additionally, 6 weeks p.i., five mice from each group were sacrificed, their spleens were separated aseptically, and their splenocytes were grown and infected by RB51 and the RB51 recombinant containing mLLO-BAX-SMAC to determine IFN-γ production [33]. B. abortus RB51 and the RB51 recombinant containing mLLO-BAX-SMAC were suspended in PBS, washed thrice, and resuspended in PBS with a concentration of 10^8^ CFU. For heat inactivation, the bacterial suspension was incubated in a 65 °C water bath for 60 min. To confirm complete inactivation, aliquots of the resulting bacterial suspensions were spread onto TSA plates and incubated at 37 °C for five days [34]. Splenocytes from the inoculated mice were obtained as previously described and grown in 10^8^ heat-inactivated *B. abortus* RB51 and RB51 recombinant containing mLLO-BAX-SMAC per well in the related groups. The cells were grown for 5 days, and their supernatants were collected. The sera and collected supernatants were tested for IFN-γ, with recombinant mouse IFN-γ as the standard. The assays were performed in triplicate [30].

### 2.11. Statistical Analyses

The counts of bacterial CFU in the cell cultures and spleens of mice were analyzed using the Mann–Whitney U test. The apoptosis, LDH, and IFN-γ production results were analyzed using one-way ANOVA. Values of *p* < 0.05 were considered significant.

## 3. Results

### 3.1. Confirmation of Transformation: Characterization of Recombinant RB51 Strains Expressing LLO and Mutant LLO-BAX-SMAC

To generate an RB51 recombinant vaccine with enhanced efficacy against bovine brucellosis, RB51 was transformed with the LLO and mutant LLO-BAX-SMAC mouse apoptotic proteins that mediate bacterial escape from the phagosome of infected cells. Hemolytic activities of LLO secreted by both RB51 recombinant strains on sheep blood agar plates were considerably more intense than those seen with the parental RB51 (Figure S1 supplemental). Furthermore, Western blot results with the his-tag antibody revealed the BAX-SMAC and LLO proteins (Figure S2 supplemental).

### 3.2. Lactate Dehydrogenase (LDH) Release

**Figure 1:**
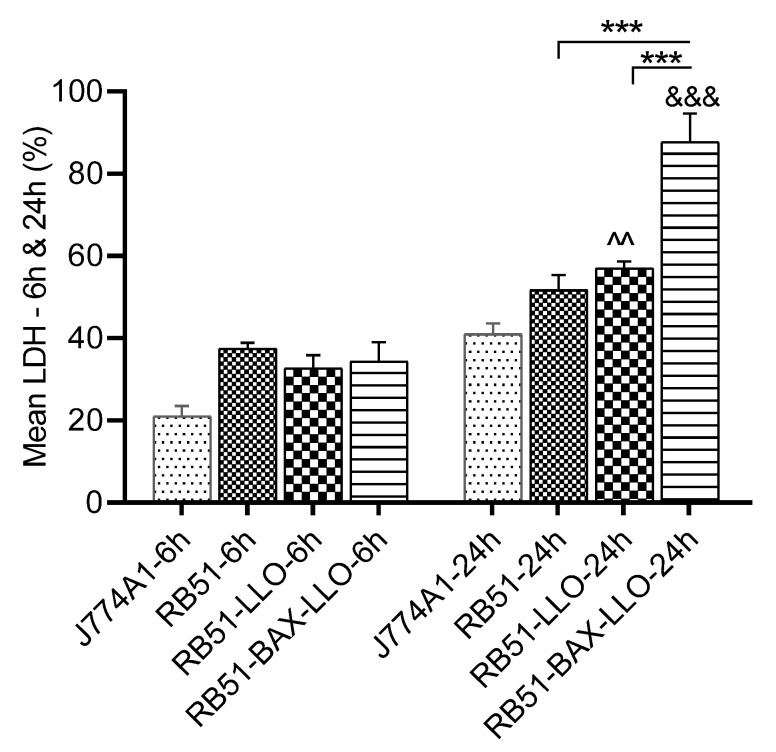
LDH release as a cytotoxicity measure induced by RB51-mLLO-BAX-SMAC-, RB51-LLO-, or RB51-infected macrophages. Six replicates were used in each of the independent experiments. The average of the three independent experiments showed no significant difference between the LDH release of cells infected with both RB51 recombinant strains and RB51-infected cells after 6 h. However, there was a significant difference between them after 24 h. ^ Significant difference compared to RB51-LLO-6 h; & significant difference compared to RB51-BAX-LLO-6 h. The data represent the means ± SEM from three independent experiments. (***^, &&&^ *p* < 0.001), (^^^^ *p* < 0.01).

Lactate dehydrogenase release in the supernatant of the infected J774A cells was measured as a means of evaluating the cytotoxic activity of the recombinant and parental RB51 strains. The results show no significant differences in LDH levels 6 h post-infection between RB51 recombinant-infected macrophages and those infected with RB51. However, after 24 h, the cytotoxicity of cells infected with both RB51 recombinants significantly increased compared to those infected with the parental RB51 (Figure 1; *p* < 0.001). Additionally, the cytotoxicity of RB51-mLLO-BAX-SMAC-infected cells significantly increased compared to those infected with RB51-LLO (*p* < 0.001).

### 3.3. Evaluation of Brucella Survival

The survival of both RB51 recombinant strains or RB51 bacteria in J774A.1 macrophage cells was evaluated by determining *Brucella* colony-forming units of infected macrophages. In addition, the presence of plasmids in recombinant strains separated from cell lines was revealed by an antibiotic resistance test. 

**Figure 2:**
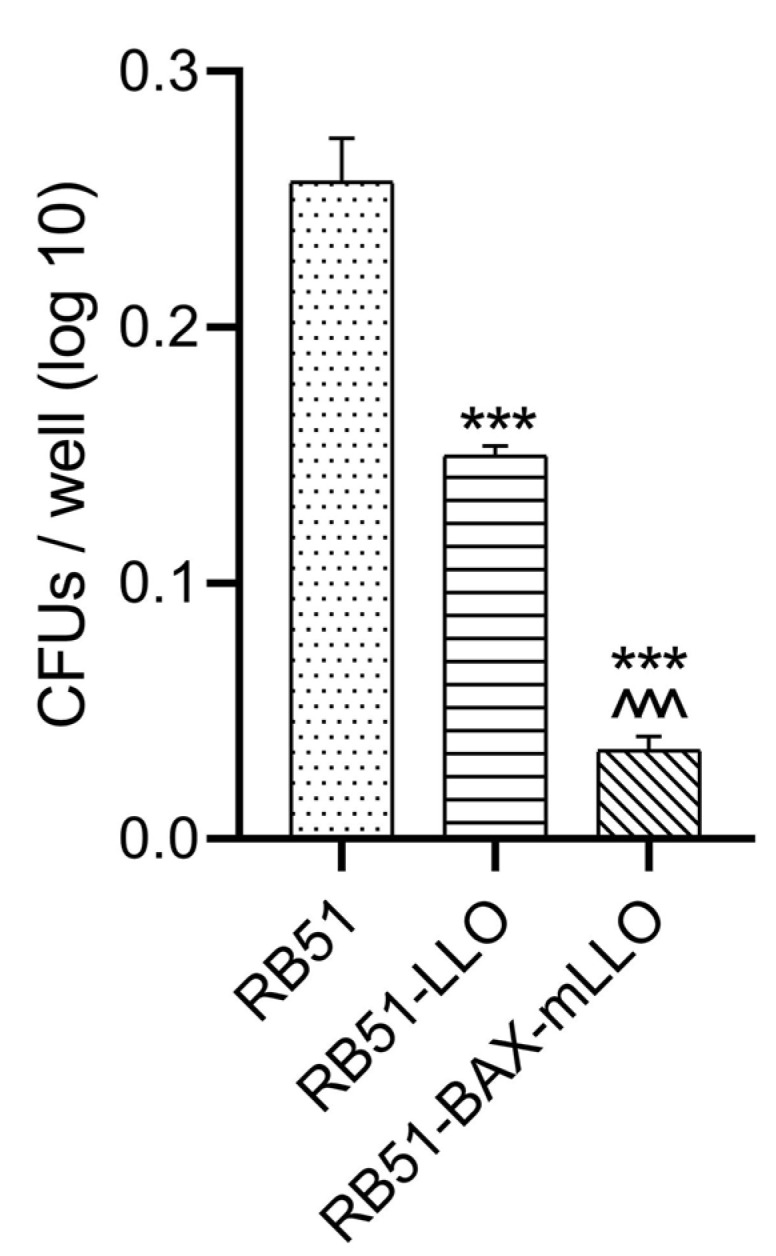
The survival rate of RB51mLLO-BAX_SMAC bacteria in J774A.1 macrophages was significantly reduced compared to RB51-LLO and RB51 bacteria. * Significant difference compared to the control (RB51) group. ^ Significant difference compared to RB51-LLO. The data represent the means ± SEM from three independent experiments. (***^, ^^^^ *p* < 0.001).

After 24 h p.i., the intracellular survival rate of both RB51 recombinant strains was significantly reduced. The RB51mLLO-BAX_SMAC bacteria in J774A.1 macrophages were significantly reduced in comparison with RB51-LLO and RB51 bacteria (*p*-value < 0.001; Figure 2).

### 3.4. Programmed Macrophage Cell Death

**Figure 3:**
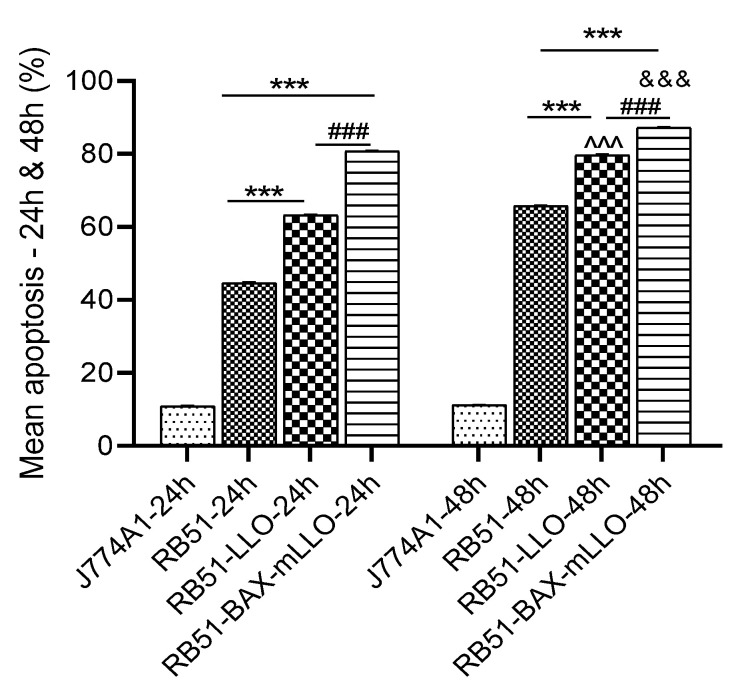
The apoptosis pattern of infected macrophages was determined by flow cytometry. Six replicates were used in each of the three independent experiments. The average of the three independent experiments showed that RB51-BAX-mLLO induced significantly higher programmed cell death than RB51-LLO and RB51 after 24 and 48 h p.i. * Significant difference compared to the control group (RB51) at each time point. # Significant difference compared to RB51-LLO at each time point. ^ Significant difference compared to RB51-LLO-24 h. & Significant difference compared to RB51-BAX-mLLO-24 h. (***^, ^^^, &&&, ###^ *p* < 0.001).

A previous study had shown that RB51 could induce apoptosis in infected macrophages [35]. However, the flow cytometry results show that J774A.1 macrophages infected with both RB51 recombinant strains induced significantly higher programmed cell death than those infected with the parental RB51 after 24 and 48 h p.i. (*p*-value < 0.001; Figure 3).

### 3.5. Animal Testing for Clearance Experiments

**Figure 4:**
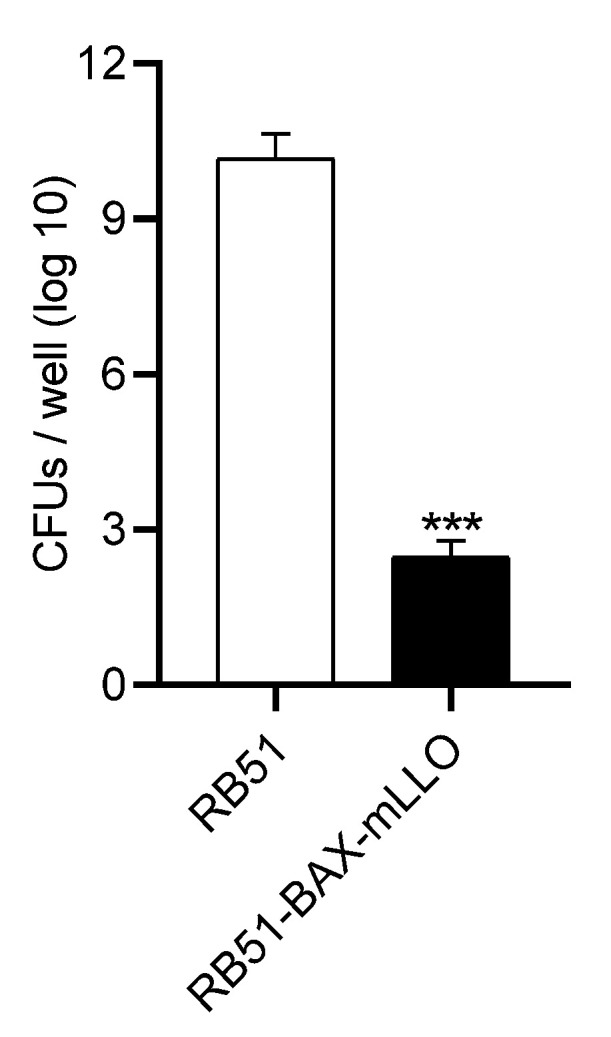
Clearance of *Brucella* in mice vaccinated with strains RB51-BAX-mLLO and RB51. Three weeks after vaccination, the number of *Brucella* CFU in the spleen was determined. There was a significant difference between the number of *Brucella* CFU in the spleen of mice vaccinated with RB51-BAX-mLLO and RB51 (*** *p* < 0.001).

To determine the increased apoptotic rates of the infected cells that could potentially lead to fast clearance of organisms from animal tissues, we determined the bacterial load recovered from the spleens at specific times after infection was determined after 21 days. The presence of plasmids in the recombinant strain separated from the spleen was revealed by an antibiotic resistance test and extraction. Mice vaccinated with the RB51-BAX-mLLO strain had lower bacterial numbers in their spleens compared to those immunized with RB51 (*p* < 0.001), indicating improved clearance and higher attenuation levels (Figure 4).

### 3.6. Cytokine Quantitation in Immunized Mice

**Figure 5:**
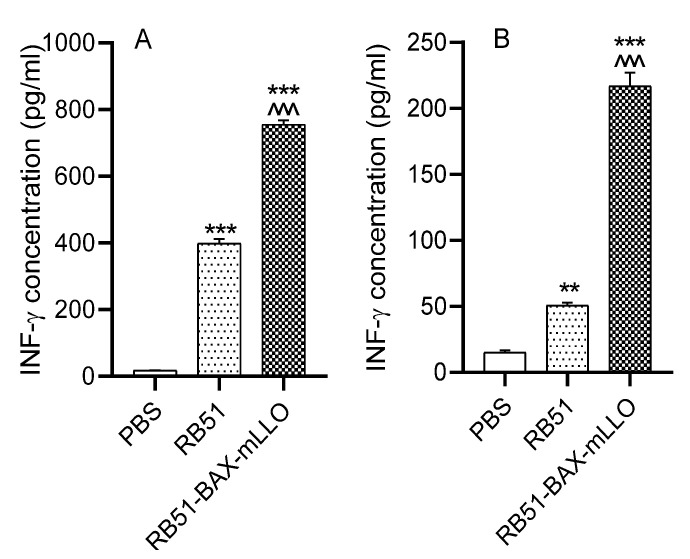
Concentration of IFN-γ in mouse sera (**A**) and culture supernatants of splenocytes upon in vitro (**B**) stimulation with heat-inactivated RB51 after 3 and 6 weeks p.i. In both cases, five replicates were used in each of the three independent experiments. * Significant difference compared to the control group (PBS). ^ Significant difference compared to the parental RB51. (***^, ^^^^ *p* < 0.001), (** *p* < 0.01).

One of the main objectives of this study was to assess the effect of RB51-mLLO-BAX-SMAC on IFN-γ overexpression as an enhanced Th1 response. Thus, vaccinated mice were monitored for IFN-γ expression. Mouse sera were collected 3 and 6 weeks after RB51-BAX-mLLO and RB51 vaccination and were used for measurement of IFN-γ production. RB51-BAX-mLLO-vaccinated mice showed up-regulation of IFN-γ (*p* < 0.001; Figure 5A). This observation was consistent with the IFN-γ analysis performed with culture supernatants from RB51-BAX-mLLO- or RB51-infected macrophages. In addition, the splenocytes from RB51-BAX-mLLO-vaccinated mice produced significantly higher levels of IFN-γ when stimulated with heat-inactivated RB51-BAX-mLLO in vitro compared with the RB51 and saline-inoculated groups, confirming the potential of the RB51 recombinant carrying mLLO-BAX-SMAC as a candidate vaccine (*p* < 0.001; Figure 5B).

## 4. Discussion

Cell-mediated immunity responses play a critical role in resistance against intracellular bacterial infections. Live attenuated vaccines are generally considered vaccines of choice for intracellular bacteria, such as *Brucella* spp. [36]. In order to enhance the efficacy of available vaccines against *Brucella*, an RB51 vaccine armed with the LLO and BAX-SMAC proteins was constructed. The phagosomal escape of the RB51 vaccine using the cytolytic activity of LLO represents a unique mechanism to facilitate antigen presentation to the immune system and apoptosis induction [14,37]. Our results demonstrate that the efficacy of the recombinant vaccine RB51-mLLO-BAX-SMAC was significantly increased. Based on confirmed tests by Kaufmann et al. (2005) on *Mycobacterium Bovis bacilli* *Calmette-Guérin* (*BCG*), it seems that membrane perforation using LLO secreted by the recombinant RB51 and LLO-BAX-SMAC facilitates the delivery of the pathogen antigens into the cytoplasm and is then processed by the proteasome and presented by the MHCI [38]. Phagolysosomal proteases also leak into the cytosol, cleave the caspases, and induce apoptosis [38]. Moreover, the RB51 recombinant-induced programmed cell death may release apoptotic vesicles that contain *Brucella* antigens and activate dendritic cells and CD8+ T cells. This process of CD8+ T cell activation is called “cross-priming.” The cross-priming process presents antigens acquired from the outside of the cell. It is hypothesized that the RB51 recombinant activates CTL activity with cross-priming [38]. Our results demonstrate that recombinant *Brucella* strains armed with both the LLO and BAX-SMAC apoptotic proteins are more enhanced than strains equipped with only LLO. It seems that phagosome membrane perforation by LLO causes BAX-SMAC proteins to leak into the cytosol, which has a binary effect on apoptosis induction. Kaufmann et al. (2005) constructed a recombinant *BCG* vaccine that secreted LLO and concluded that the LLO-secreting r*BCG* strain offered more efficacious protection against *M. tuberculosis* than the parental *BCG*. This improvement was primarily based on better cross-priming, which causes enhanced T cell-mediated immunity in mice. Furthermore, LLO promoted antigen translocation into the cytoplasm and apoptosis of infected macrophages [38].

LLO has a pronounced acidic optimum pH that helps *Listeria* escape from acidic phagosomes and spread in the cytosol. However, available evidence suggests that at the early stages of *Brucella* phagocytosis, inhibition of phagosome acidification by the urease activity of *Brucella* can modify the phagosomal pH to remain neutral, a condition that is not suitable for LLO activity [39]. Based on past studies, the change of leucine 461 to threonine increased the hemolytic activity of LLO almost 10-fold at neutral pH [22]. We, therefore, transformed the RB51 strain with a vector containing the mutant LLO L461T to improve its activity. Leucine 461 is responsible for the optimum acidic pH of LLO, and the change of leucine 461 to threonine significantly increased the lytic activity of LLO in the RB51 recombinant compared to the parental RB51. Mycobacterial urease C has an essential role in pH neutralization of the host phagosome and thus inhibits the maturation of the phagolysosome. To improve the pH for LLO activity, a urease C-deficient r*BCG* strain that lacked urease activity was constructed by Kaufmann et al. They showed that LLO secreted by urease C-deficient *BCG* was more active than that secreted by *BCG* armed with LLO without urease deletion because of an optimized phagosomal pH for LLO [14].

This report investigated the immune response elicited by the RB51 recombinant containing mLLO-BAX-SMAC compared with the parental RB51. Mice vaccinated with strain RB51 carrying mLLO-BAX-SMAC developed an enhanced Th1-type immune response mediated by the secretion of IFN-γ in the serum and supernatant of stimulated splenocytes. The Th1-type immune response plays an essential role in protection against *Brucella*. Therefore, alteration in the processing and presentation of *Brucella* antigens may cause a protection enhancement.

One disadvantage of existing *Brucella* live attenuated vaccines such as RB51 is the incomplete immunization clearance from animal tissue, and persistent infections with vaccine strains have occasionally been reported in vaccinated animals [8]. One of the desired features of our candidate vaccine is prompt clearance from the spleen as a measure of potency and safety. Our finding reveals that the clearance patterns of the recombinant strain and parental RB51 in the vaccinated mice were not similar. However, the clearance of the RB51 recombinant containing mLLO-BAX-SMAC was better than that of the parental RB51. It seems that the high rate of apoptosis in the infected spleen cells and the effective immune response led to better clearance of bacteria from the mouse tissue. Furthermore, the increased LDH activity of our recombinant strain in the infected cell line compared to the parental strain may result from lysis of the macrophages or intracellular killing activity that may help explain the decline in the number of recombinant vaccine organisms in the mouse spleen tissue.

Additionally, the recombinant strains had a lower survival rate in macrophages while maintaining their attenuation characteristic.

In conclusion, past studies reported that the parental RB51 poorly stimulates the CTL response by CD8 T cells and Th1; however, here, we demonstrated that RB51 strains expressing LLO and BAX-SMAC promoted a robust Th1-type immune response. As a limitation, we did not challenge the mice with a *Brucella* abortus wild type. Future experiments should be directed at clarifying whether these recombinant strains possess increased vaccine efficacy against *Brucella* in animal models. 

## Data Availability

Not applicable.

## Supplementary Materials

The following are available online at https://www.mdpi.com/article/10.3390/vaccines10030388/s1. Figure S1. Hemolytic activities of LLO secreted by rRB51 strains on sheep blood agar plate. Figure S2. Western blots showing secretion of mLLO and BAX-SMAC by rRB51. Lane 1, RB51 culture supernatant; Lanes 2, 3, 4, and 5, the supernatant of rRB51 culture; Lane 6, Protein marker. The 21kDa and 58kDa bands are related to BAX-SMAC and mLLO protein, respectively.
