# Supplementary material for: Design and Characterization of a Recombinant Brucella abortus RB51 Vaccine That Elicits Enhanced T Cell-Mediated Immune Response
Source: Vaccines (Basel). 2022 Mar 3;10(3):388. doi: 10.3390/vaccines10030388 (PMC8950781; doi:10.3390/vaccines10030388)
Supplement: Supplementary file 1 [file vaccines-10-00388-s001.zip › vaccines-1506508-supplementary.pdf]

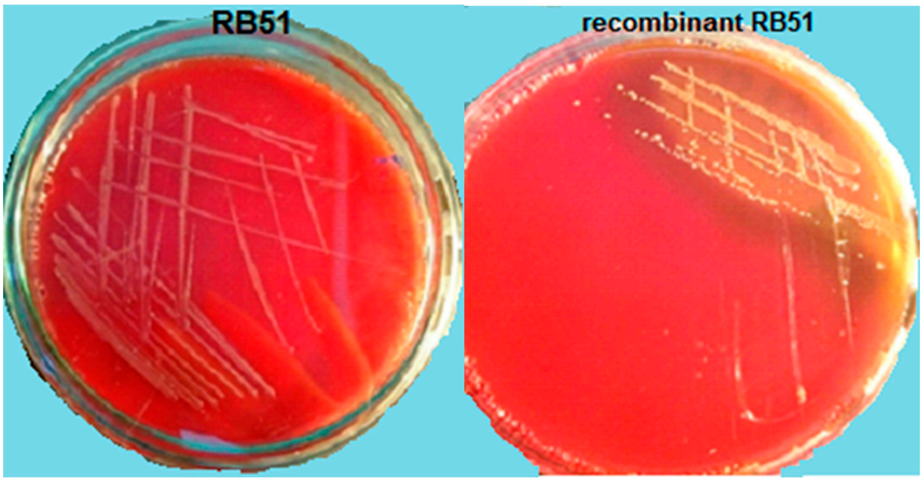

Figure S1. Hemolytic activities of LLO secreted by rRB51 strains on sheep blood agar plate.

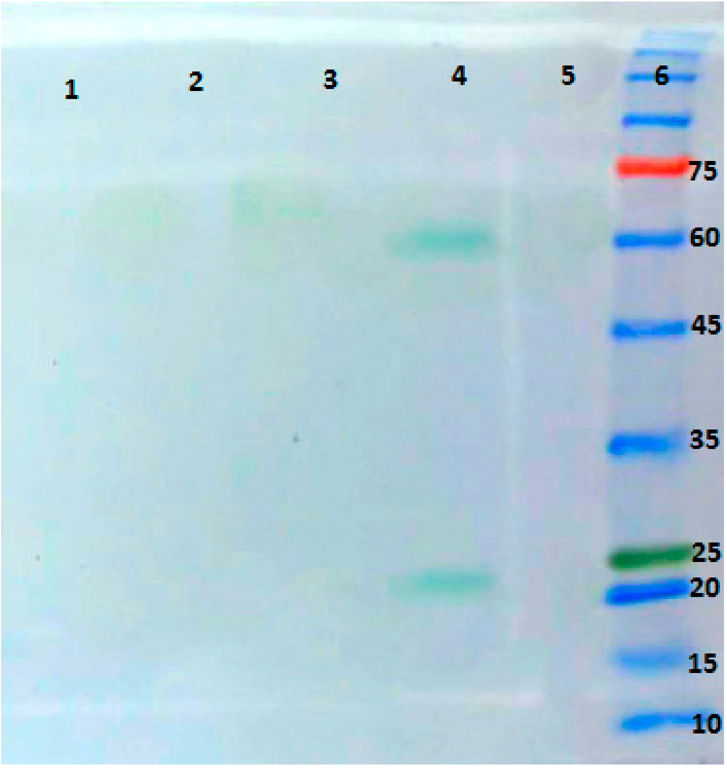

Figure S2. Western blots showing secretion of mLLO and BAX-SMAC by rRB51. Lane 1, RB51 culture supernatant; Lanes 2, 3, 4, and 5, the supernatant of rRB51 culture; Lane 6, Protein marker. The 21kDa and 58kDa bands are related to BAX-SMAC and mLLO protein, respectively.
